# Effects of a dietary supplement on golf drive distance and functional indices of golf performance

**DOI:** 10.1186/s12970-014-0065-4

**Published:** 2015-01-21

**Authors:** Tim N Ziegenfuss, Scott M Habowski, Robert Lemieux, Jennifer E Sandrock, A William Kedia, Chad M Kerksick, Hector L Lopez

**Affiliations:** The Center for Applied Health Sciences, 4302 Allen Road, Ste 120, Stow, OH 44224 USA; Strength and Conditioning, Athletics Department, Kent State University, Kent, OH 44240 USA; School of Sport, Recreation and Exercise Sciences, Lindenwood University, St. Charles, MO 63301 USA

**Keywords:** Golf, Performance, Creatine, Drive distance, Velocity, Driver

## Abstract

**Background:**

Limited research exists examining the impact of nutrition on golfing performance. This study’s purpose was to determine the impact of daily supplementation with an over-the-counter dietary supplement on golf performance.

**Methods:**

Healthy men (30.3 ± 6.9 y, 183.1 ± 5.6 cm, 86.7 ± 11.9 kg), with a 5–15 handicap were assigned in a double-blind, placebo-controlled manner to ingest for 30 days either a placebo (PLA, n = 13) or a dietary supplement containing creatine monohydrate, *coffea arabica* fruit extract, calcium fructoborate and vitamin D (Strong Drive™, SD, n = 14). Subjects ingested two daily doses for the first two weeks and one daily dose for the remaining two weeks. Participants followed their normal dietary habits and did not change their physical activity patterns. Two identical testing sessions in a pre/post fashion were completed consisting of a fasting blood sample, anthropometric measurements, 1-RM bench press, upper body power and golf swing performance using their driver and 7-iron. Data were analyzed using two-way mixed factorial ANOVAs and ANCOVA when baseline differences were present. Statistical significance was established *a priori* at p ≤ 0.05.

**Results:**

ANCOVA revealed significantly greater (post-test) best drive distance (p = 0.04) for SD (+5.0% [+13.6 yards], ES = 0.75) as well as a tendency (p = 0.07) for average drive distance to increase (+8.4% [+19.6 yards], ES = 0.65), while no such changes were found with PLA (−0.5% [−1.2 yards], ES = 0.04 and +1.3% [+2.8 yards], ES = 0.08, respectively). Both groups experienced significant increases in body mass and 1-RM bench press (p < 0.001). No other significant group × time interactions were found. For the SD group only, within-group analysis confirmed significant improvements in set 1 average (+8.9%, p = 0.001) and peak velocity (+6.8%, p < =0.01). No changes were noted for reported adverse events, pain inventories, quality of life or any measured blood parameter.

**Conclusions:**

SD supplementation for 30 days significantly improved best drive distance more than placebo. Supplementation was well tolerated and did not result in any clinically significant changes in markers of health or adverse events/side effect profiles.

## Introduction

The sport of golf requires an intricate mix of physical, emotional and cognitive factors to achieve optimal performance [1]. The swing itself is a complex pattern of coordinated biomechanical movements that impact both the accuracy and distance with which the ball is struck. In its purest sense, the golf swing is a movement centered mostly upon the production of power; consequently, swings are considered to be largely anaerobic in nature [1].

As the popularity of golf rises, the development of ergogenic approaches have produced a seemingly endless array of clubs and other pieces of equipment intended to help improve performance. In light of the physical and cognitive challenges brought forth by golf, nutritional approaches may impact performance, but minimal effort, to date, has been made in this area.

The lack of nutritional considerations within the sport of golf is somewhat surprising. Currently, nutritional guidelines center upon prudent management of fluid and carbohydrate levels, but other demands found in golf present the need for additional nutritional concerns. For example, under certain environmental conditions the extended duration to complete a round of golf (~3 – 4 hours) can promote dehydration, reduced energy levels and mental fatigue [1]. When these factors are considered collectively, it makes intuitive sense that a nutritional formulation that can improve anaerobic performance and increase a golfer’s focus and attention may impact performance. In this respect, one of the only published studies to examine the impact of a nutritional agent on golfing performance utilized phosphatidylserine supplementation to lower the stress hormone cortisol and improve shot-making during simulated golf swings [2]. SuperDrive™ (Purity Products, Plainview, NY) is a commercially available dietary supplement that combines creatine monohydrate, coffee arabica fruit extract, calcium fructoborate and vitamin D and is marketed to the golfing community to aid in performance and recovery from musculoskeletal complications associated with participation in golf [1].

Creatine monohydrate is one of the most popular and effective dietary supplements due to its ability to improve strength, power, lean mass and explosive performance. Creatine supplementation protocols are well established to significantly increase intramuscular levels of phosphocreatine and total creatine which, in the vast majority of studies, result in measureable improvements in a wide variety of high-intensity activities [3–8]. However, to our knowledge, no research is available that has examined the impact of creatine supplementation on golfing performance. In light of the fact that the golf swing is an anaerobic event [1], it is logical that creatine supplementation may have the potential to improve golf swing power.

*Coffea arabica* is a fruit extract (CoffeeBerry®) that is derived from the same plant as traditional coffee, thus making caffeine the primary active ingredient found within the extract. For years, scientific research has supported the use of caffeine as an ergogenic aid, primarily known for its positive impact on executive functions such as focus, attention and concentration, stimulation of fatty acid mobilization, improvements in endurance performance [9], and various direct effects on muscle function [10,11]. From a physical perspective, caffeine is known to antagonize adenosine receptors, thereby inhibiting the negative impact of adenosine on neurotransmission, arousal, and pain perception [12] in addition to reducing ratings of perceived exertion [13]. Beyond physical benefits, caffeine can also operate to enhance a number of cognitive aspects. Caffeine is commonly used and accepted for its ability to promote wakefulness as well as enhance focus and concentration, all attributes that would be of particular benefit to a golfer, while also preventing both peripheral and central manifestations of fatigue [10]. An excellent review by Glade summarized available literature on caffeine’s impact of cognitive functioning and reported that caffeine in doses in modest amounts (30 – 50 mg) are able to favorably impact mental energy, but typical doses of 100 – 150 mg are needed to positively impact assessments of cognitive functioning [10]. Similarly, Einother and investigators expertly summarized the literature and concluded that caffeine favorably impacts both simple and complex tasks through both attention enhancement and optimized executive function [14].

A myriad of musculoskeletal problems can negatively impact golfing performance and strategies to minimize pain and joint discomfort while also promoting favorable bone and muscle health are important concerns for active golfers. The trace mineral boron is closely linked to improved rates of calcium retention and bone health. Although no recommended daily allowance for boron has been established, doses ranging from 1–4 mg have been shown to safely promote improvements in bone density [15,16]. Recent technology has produced calcium fructoborate (FruiteX-B®), a patented compound that results in calcium being bound to boron, forming a natural chelation and effective stabilization of the available boron. While relatively new, calcium fructoborate has been shown to exert strong anti-inflammatory functions [17] and pronounced antioxidant activity [18]. Interestingly, the compound can favorably impact hormonal regulation and vitamin D metabolism leading to its interest as a pain and bone/joint health aid. Specifically, unpublished research in mild and severe osteoporotic patients using an open-label approach indicated that eight weeks of supplementation with calcium fructoborate reduced pain and stiffness during walking and climbing stairs and improved joint mobility [19]. More recent published literature utilized a two-week double-blind, placebo-controlled supplementation approach on 116 osteoporotic patients and concluded that calcium fructoborate supplementation improved inflammatory markers for all groups [20].

Another ingredient that continues to get attention regarding its ability to favorably impact bone health [21] and healthy muscle metabolism [22–24] is vitamin D. The average adult American diet contains only 150–300 IU of vitamin D per day while recommended levels are 600–800 IU, despite multiple studies indicating that higher daily amounts may be optimal [21,25]. Currently, the Endocrine Society recommends 1,500 – 2,000 IU/day, with other reports recommending between 400 – 1,000 IU/day. To date, the impact of supplementation with vitamin D (either in isolation or as part of a multi-ingredient formula) on golf performance is unknown.

The primary purpose of this preliminary, proof-of-concept investigation was to examine the impact of a nutritional formulation containing creatine, *coffea arabica* fruit extract (CoffeeBerry®), calcium fructoborate (FruiteX-B®) and vitamin D on functional indices of golf swing performance, and upper-body strength/power. Secondary purposes were to assess changes in indices of quality of life, pain and standard blood-based markers of clinical safety and health.

## Methods

### Overview of research design

This study was completed as a randomized, double-blind, placebo-controlled trial. Over a 30 day period, eligible study participants who first signed an IRB-approved informed consent document were assigned in a randomized, double-blind, placebo-controlled fashion to ingest either a dietary supplement or a placebo. In an identical fashion, two daily supplement (or placebo) doses were ingested during the first two weeks and one daily dose was ingested during the final two weeks for 30 days total. Prior to testing, all study participants were instructed to refrain from heavy exercise for 48 hours and observe a 12 hour fast. To enhance reliability and minimize a learning effect, subjects were familiarized to all experimental procedures prior to testing. Participants then completed two identical testing sessions consisting of a fasting blood sample, anthropometric assessments, resting heart rate and blood pressure, muscular strength and power assessments, golf swing performance, and quality of life, pain and adverse event questionnaires. To determine indications of clinical safety, fasting blood samples were collected and analyzed for complete blood counts, clinical chemistry panels and other indicators of health and safety along with resting levels of heart and blood pressure. To assess changes in strength and power and evaluate the impact of supplementation, bench press 1-RM was determined along with bench press throw power, respectively. In addition to these accepted laboratory methods of upper body strength and power, functional performance was also assessed using three-dimensional analysis of a series of golf swings using participants’ 7-iron and driver. Finally and as a general means to assess the impact of supplementation on potential changes in musculoskeletal health, self-assessments of pain, quality of life and other adverse outcomes were gleaned from all study participants.

### Subjects

Twenty-seven healthy men (mean ± SD age, height, body mass: 30.3 ± 6.9 y, 183.1 ± 5.6 cm, 86.7 ± 11.9 kg) with a handicap index of 5–15 were recruited as participants in this study. Prior to any research-related activity, all study participants reviewed and signed an IRB approved informed consent document (Integreview, Austin, TX, Protocol #PUR-002, approval date: July 30, 2013). During their first laboratory visit, participants completed medical history paperwork and were screened for eligibility by a licensed physician. Inclusion criteria for this study required study participants to be in good health as determined by medical history review and baseline blood chemistries, normotensive (systolic blood pressure ≤ 140 mm Hg, diastolic blood pressure ≤ 90 mm Hg and resting heart rate ≤ 90 beats per minute) and have maintained a modest level of physical activity (defined as an average of two workouts per week) for at least one year. Participants were excluded if they had any metabolic disorder including known electrolyte abnormalities, diabetes, thyroid disease, hypogonadism, or a history of hepatic, renal, musculoskeletal, autoimmune, or neurologic disease. Exclusion criteria also included subjects with history of heart disease, hypertension, psychiatric disorders, cancer, benign prostatic hypertrophy, gastric ulcer, gastroesophegal reflux disease, or any other medical disorder deemed unsuitable for inclusion in the study by the investigators. Participants who reported a history of taking creatine or other dietary supplements were only allowed entry if they had not taken or had refrained from taking any dietary supplements containing these ingredients for at least 30 days prior to initiating the study (excluding a multi-vitamin/mineral) and agreed to only use their assigned supplement throughout the study protocol. Individuals who reported as regularly consuming caffeine (<200 mg/day) were instructed to not change their caffeine intake throughout the duration of the protocol; participants who consumed >200 mg caffeine were excluded. Participants currently prescribed any thyroid, antihyperlipidemic, hypoglycemic, antihypertensive, anticoagulant, or androgenic medications, nitrates/nitrate derivatives, or phosphodiesterase (PDE)-5 inhibitors were also excluded. Subjects who had admitted to using anabolic steroids, growth hormone, insulin-like growth factor (IGF)-1, or other hormone medication including oral contraceptives during the previous 12 months were also excluded, as were smokers and those with orthopedic limitations or injuries.

### Procedures

#### Testing protocol

All study participants were first familiarized to all experimental procedures prior to completing their first testing session. Prior to arriving for all testing sessions, study participants were instructed to refrain from physically taxing exercise for 48 hours and to observe a 12 hour fast. Upon arrival for the initial testing session, physical activity and health history were determined using standardized questionnaires. Subjects then had their resting heart rate and blood pressure determined using an automated sphygmomanometer, standing height determined using a wall-mounted stadiometer, and body mass determined using a calibrated scale (Seca Medical Scale, Hanover, MD). On separate days thereafter, study participants completed a 1-RM test on the bench press, an upper-body power test (bench press throws) and an assessment of their golf swing performance.

#### Supplementation protocol

**Figure 1:**
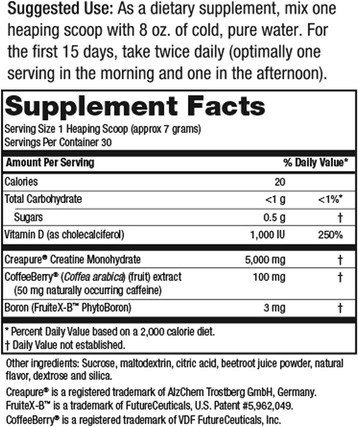
**Supplement Facts for Strong Drive™.**

In a double-blind, placebo-controlled fashion, study participants were instructed to ingest either a powdered dietary supplement containing creatine, *coffea arabica* fruit extract (CoffeeBerry®), calcium fructoborate (FruiteX-B®) and vitamin D (Strong Drive™, SD, n = 14) or an isocaloric placebo (PLA, n = 13). A representative Supplement Facts label of the investigational product is shown in Figure 1. Irrespective of group assignment, all study participants were instructed to take each serving of their assigned supplement with eight ounces of cold water. For the first two weeks of the study, one serving was consumed twice per day (with breakfast and lunch). During the final two weeks of the study, study participants consumed only one serving per day of their assigned supplement (with breakfast). This protocol was employed to match manufacturer guidelines; the protocol also corresponded with previous creatine supplementation literature showing effective increases in intramuscular creatine and phosphocreatine levels [3,6]. To ensure complete blinding, all study supplements were in powder form of similar color, texture and flavor while also being packaged in coded generic containers. Compliance to the supplementation protocol was monitored by having study participants complete a supplementation log. In addition, study participants were required to return their empty supplement containers and were reminded of details associated with the study protocols with weekly text messages and/or emails. Participants were instructed to refrain from using other supplemental courses of caffeine or creatine.

#### Blood collection and analysis

Whole blood and serum samples were collected using standard phlebotomy techniques on day 0 and day 30 of the study protocol. Whole blood samples were collected into K2-EDTA treated vacutainer tubes and upon collection were slowly inverted ten consecutive times prior to immediate refrigeration. Serum samples were collected in non-treated tubes and allowed to clot for 30 minutes at room temperature prior to being centrifuged (Horizon mini E Centrifuge, Drucker Diagnostics, Port Matilda, PA) for 15 minutes at 3200 rpm. Serum was extracted from all samples and aliquots were pipetted into cryovial storage tubes. All blood samples were analyzed for clinical chemistry analysis (plasma glucose, blood urea nitrogen [BUN], creatinine, aspartate aminotransaminase [AST], alanine aminotransaminase [ALT], creatine kinase, lactate dehydrogenase, total bilirubin, alkaline phosphatase [ALP], triacylglycerol [TG], total cholesterol [TC], LDL, HDL, uric acid, sodium, potassium, total protein, albumin, globulin, iron, complete blood cells, and platelet count) using automated clinical chemistry analyzers (LabCorp, Dublin, OH branch). All samples from the same day were batch analyzed with test-retest reliabilities commonly reported using internal quality control data from clinical laboratories and associated automated analyzers within a range of 3 – 5% [26].

#### Muscular strength and power determination

On day 0 and day 30, maximal upper body muscle strength was estimated by 1-RM bench press testing using protocols adapted from the National Strength and Conditioning Association [27]. Only upper-body strength was assessed in this study due to previous experience in our lab and others indicating greater reliability of upper-body vs. lower-body testing [28]. It is recognized that the bench press exercise acts primarily in the sagittal plane whereby the golf swing occurs through multiple planes at a wide range of accelerations and decelerations. In this respect, Marta and colleagues published a review of EMG studies involving the golf swing and they reported that the pectoralis muscle group experienced a great deal of activity during the golf swing movement [29]. Thus, our analytical approach in this styd was to combine a standardized, well-accepted measure of upper-body strength and power (1RM bench press and bench throws) along with a three-dimensional analysis of golf swing performance (described below) to assess the potential impact of nutritional supplementation. After a general warm-up of three to five minutes of light activity consisting of upper body ergometry and static stretching of the involved musculature, the subject performed a warm-up set of ten repetitions with only the bar, followed by eight repetitions at approximately 50% of their estimated 1-RM, followed by one set of three repetitions at 70% of their perceived 1-RM. Thereafter, study participants performed single repetition lifts at progressively increasing loads until 1-RM was determined. No more than five maximal attempts were completed in one testing session.

Upper body power production (average power [AP], average velocity [AV], peak power [PP], peak velocity [PV]) was assessed using the bench press exercise using a Tendo unit interfaced to a standard Smith machine rack. Previous studies have successfully incorporated the use of a Tendo into their study design [30] and Stock and colleagues [31] recently published data to indicate it is a reliable means of assessment. The unit consists of a position transducer that measures the rate of linear displacement providing velocity and acceleration in addition to power production. After 1RM determination, subjects rested for five minutes and completed three sets of three explosive repetitions (i.e. bench throws) with a load equal to 40% of their actual 1-RM on the bench press. Interset rest periods were strictly standardized at 90 seconds each. The reliability of our procedures using these procedures is similar to that previously reported [28,31].

#### Golf swing performance

On day 1 and day 30, golf swing performance was measured using a three-dimensional (3-D) swing analysis system (TRACKMAN PRO, Brighton, MI) by a NCAA Division I golf coach. Study participants completed a series of 10 successive swings using their 7-iron and driver. Although a wide variety of data is captured with this system (e.g. club delivery, ball launch, flight data) for the purpose of this study we chose to focus on peak and average club head speed, ball speed, as well as average and best distance for each club. Reliability was determined by having five representative participants complete five swings using both the 7-iron and driver clubs. Intraclass correlation coefficient and standard error of measurement using the 7-iron were calculated for ball speed (ICC_3,1_: 0.934, SEM: 10.99 miles/hour), club speed (ICC_3,1_: 0.991, SEM: 2.42 miles/hour) and total distance (ICC_3,1_: 0.862, SEM: 41.74 yards). Identical measurements were made using the driver for ball speed (ICC_3,1_: 0.975, SEM: 3.26 miles/hour) , club speed (ICC_3,1_: 0.990, SEM: 2.28 miles/hour) and total distance (ICC_3,1_: 0.967, SEM: 8.46 yards). All calculations and assumptions were made according to Weir 2005 [32].

#### Qualitative assessments

Study participants were asked to maintain their normal patterns of exercise and habitual physical activity. Assessment of physical activity was completed on day 0, day 1 and day 30 using the Yale Physical Activity Survey prior to the study protocol and again on the last day of testing [33]. The Brief Pain Inventory and Quality of Life (SF-12, version 2) were also administered on day 1 and day 30.

#### Dietary intake

No dietary restrictions or prescriptions were made as part of this study protocol. Subjects recorded their dietary intake over a three day period (two week days, one weekend day) according to instructions given by a research dietitian on day 0 and day 30. Each subject’s baseline diet was analyzed by NutriBase IX software (CyberSoft, Inc., Phoenix, AZ) to determine average energy and macronutrient content as well as distribution. Additional three-day dietary records were collected and analyzed in an identical fashion at the end of the supplementation protocol (day 30). Twenty-four hours prior to post-testing on day 30, study participants duplicated their dietary intake using diet records from day 0.

### Statistical analyses

Data were analyzed using a two-way mixed factorial ANOVA (treatment [PLA vs. SD] x time [pre vs. post]) with repeated measures on the time factor. ANCOVA was utilized when baseline differences were present using respective baseline scores as the covariate. Within-group main effects over time were fully decomposed using paired samples t-tests. Within-group effect sizes (ES) were also calculated (post mean – pre mean/pooled SD) for all variables and are reported in table format. Normality was determined on all data using the Shapiro-Wilk statistics and visual inspection of standardized skewness and kurtosis scores. When the sphericity assumption was not met, the Huynh-Feldt correction was applied. Any non-normally distributed data were log-transformed prior to analysis. A p-value of less than or equal to 0.05 was used for determination of statistical significance. Trends were identified as p-values between 0.051 and 0.10. All statistical analyses were completed using SPSS software, version 21 (Cary, NC).

## Results

Of the 45 people screened for this study, 30 met inclusion criteria and were randomized to the study protocol. Reasons for excluding participants included not achieving an adequate golf handicap score, taking medications for blood pressure and not wanting to cease current supplement use. Of the 30 people randomized into the protocol, three participants were dropped because one did not arrive for post-testing while two others failed to show for their final golf swing performance test. Thus, 27 people completed the study. Descriptive characteristics and baseline demographics are found in Table 1. No significant differences (p > 0.05) were found at baseline between groups for age, height, body mass, systolic blood pressure, or diastolic blood pressure. Resting heart rate values at baseline tended to be greater in the PLA group (PLA: 67.5 ± 8.4 vs. SD: 61.4 ± 7.4 beats/min, p = 0.052). In response to the intervention, no significant group × time interaction (p > 0.05) effects were revealed for body mass, systolic blood pressure, diastolic blood pressure or resting heart rate.

**Table 1:** **Baseline anthropometric and hemodynamic characteristics of study participants**

	**PLA (n = 13)**	**SD (n = 14)**	**Between groups (** ***p*** **)**
Age (years)	30.1 ± 7.9	30.5 ± 6.0	0.876
Height (cm)	182.1 ± 5.4	184.0 ± 5.7	0.392
Body mass (kg)	85.4 ± 9.0	87.8 ± 14.4	0.601
Systolic BP (mm Hg)	133.3 ± 15.0	129.6 ± 11.0	0.464
Diastolic BP (mm Hg)	81.5 ± 6.5	80.0 ± 6.9	0.556
Resting HR (beats/min)	67.5 ± 8.4	61.4 ± 7.4	0.052
1RM (kg)	85.1 ± 20.9	105.5 ± 25.0	0.031*

Values are mean ± SD. *Significant difference between groups via t-test, p < 0.05.

Data from maximal strength and power assessments are found in Table 2. Significant baseline differences (p < 0.05) were found for maximal strength and peak power production during all sets of the bench throws. As a result, ANCOVA was used to determine statistical differences for these variables during post-testing. Both the PLA and SD groups experienced similar significant increases in body mass (data not shown) and 1RM bench press across time (p < 0.001 in both groups) with but no between-group differences were present via ANCOVA (p = 0.86). Using ANCOVA, no between group differences (p > 0.05) were found for peak power production during all sets that were completed (Table 2). Using 2x2 mixed factorial ANOVA, no significant group × time interaction were found for peak velocity during set 2 and set 3 while the peak velocity tended (p = 0.07) to change for set 1 peak velocity (Table 2). Additionally, the SD group experienced a significant within-group increase (delta: 0.079 ± 0.089 miles/hour, p = 0.005; ES = 0.62) in peak velocity during the 1^st^ set of bench throws. In contrast, within-group changes in the PLA group for peak velocity during set 1 of bench throws were not significant (delta: 0.015 ± 0.086, p = 0.55; ES = 0.11).

**Table 2:** **Upper-body maximal strength and power characteristics**

	**Intervention**			**Comparison of change (p)**		
	**Pre**	**Post**	**Change**	**Effect size (** ***d*** **)**	**Within groups**	**Group × Time**
Bench press 1RM (kg)						
PLA	85 ± 21	89 ± 22	3.8 ± 3.0	0.18	<0.01*	0.86^§^
SD	106 ± 25†	109 ± 25	3.7 ± 2.9	0.15	<0.01*	
Bench Press Throws Peak Power Set 1 (watts)^‡^						
PLA	396 ± 91	410 ± 105	14.5 ± 43.8	0.16	0.26	0.35^§^
SD	487 ± 124	517 ± 124	28.6 ± 26.7	0.23	<0.01*	
Bench Press Throws Peak Velocity Set 1 (m/s)^‡^						
PLA	1.20 ± 0.13	1.22 ± 0.12	0.015 ± 0.086	0.11	0.55	0.07
SD	1.17 ± 0.13	1.25 ± 0.11	0.079 ± 0.089	0.62	0.02*	
Bench Press Throws Peak Power Set 2 (watts)^‡^						
PLA	401 ± 86	422 ± 108	21.4 ± 44.7	0.25	0.11	0.36^§^
SD	504 ± 124	513 ± 129	8.7 ± 38.8	0.07	0.42	
Bench Press Throws Peak Velocity Set 2 (m/s)^‡^						
PLA	1.21 ± 0.10	1.24 ± 0.16	0.025 ± 0.132	0.26	0.52	0.86
SD	1.22 ± 0.15	1.24 ± 0.10	0.016 ± 0.096	0.11	0.53	
Bench Press Throws Peak Power Set 3 (watts)^‡^						
PLA	400 ± 95	416 ± 97	15.5 ± 36.8	0.16	0.15	0.75^§^
SD	515 ± 118	523 ± 126	8.5 ± 40.3	0.07	0.44	
Bench Press Throws Peak Velocity Set 3 (m/s)^‡^						
PLA	1.21 ± 0.10	1.24 ± 0.12	0.032 ± .109	0.31	0.31	0.61
SD	1.25 ± 0.14	1.27 ± 0.09	0.012 ± 0.093	0.08	0.63	

Values are mean ± SD. ^‡^= 3 sets × 3 reps of bench press throws @ 40% 1RM. § = Group differences assessed using ANCOVA with respective baseline scores as the covariate due to significant difference between groups at baseline. *= Significant within group change.

**Figure 2:**
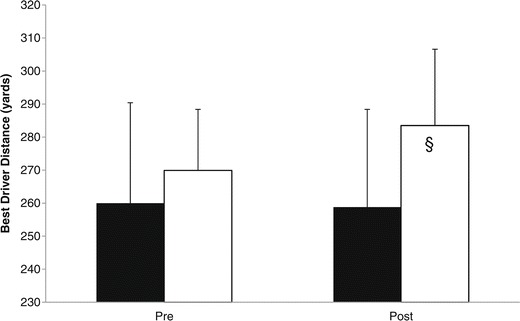
**Best distance using driver for placebo = PLA (black bars) and Strong Drive = SD (white bars).** § = Significant group differences using ANCOVA (p = 0.04) with respective baseline scores as the covariate due to significant differences between groups at baseline.

Golf performance data are shown in Table 3. Using 2×2 mixed factorial ANOVA, no significant (p > 0.05) group × time interaction effect was found for best 7-iron club speed, best 7-iron ball speed and best 7-iron distance. There was a significant difference at baseline (p < 0.05) in best driver distance, best driver club speed, and best driver ball speed, thus ANCOVA was used to compare post-test values for these variables. No between-group differences were noted for best driver club speed and best driver ball speed, but a significant difference was found for best driver distance (Table 3 and Figure 2) and there was a tendency for average driver distance to be different (PLA: 2.7 ± 25.2 vs. SD: 13.6 ± 24.5 yards, p = 0.07). In the SD group, a trend (delta: 13.6 ± 29.0 yards, p = 0.10, ES = 0.75) was identified for best drive distance. No within-group changes were seen in the PLA group for any of the golf performance parameters with the exception of a tendency for an increase in best 7-iron distance (+6.9 ± 13.2 yards, p = 0.08, ES = 0.31).

**Table 3:** **Golf performance using repeated measures ANOVA**

dummyonly	**Intervention**			**Comparison of change (p)**		
	**Pre**	**Post**	**Change**	**Effect size (** ***d*** **)**	**Within groups**	**Group × Time**
Best 7 Iron Club Speed (miles/hour)						
PLA	86.7 ± 8.9	88.0 ± 4.6	1.39 ± 4.9	0.15	0.32	0.50
SD	92.1 ± 4.8	92.5 ± 5.2	0.44 ± 1.7	0.09	0.34	
Best 7 Iron Ball Speed (miles/hour)						
PLA	113.8 ± 12.9	115.4 ± 8.8	1.51 ± 7.5	0.11	0.79	0.44
SD	120.8 ± 6.3	120.5 ± 6.9	−0.25 ± 3.9	0.04	0.80	
Best Distance 7 Iron (yards)						
PLA	162 ± 24	169 ± 21	6.9 ± 13	0.31	0.08	0.93
SD	174 ± 15	181 ± 13	7.4 ± 18	0.53	0.14	
Best Driver Club Speed (miles/hour)						
PLA	104.5 ± 9.3	104.9 ± 6.5	0.45 ± 4.6	0.05	0.73	0.46^§^
SD	110.4 ± 5.1	110.1 ± 5.7	−0.31 ± 3.6	0.06	0.76	
Best Driver Ball Speed (miles/hour)						
PLA	147.5 ± 13.8	148.6 ± 9.5	1.1 ± 7.5	0.09	0.61	0.17^§^
SD	158.9 ± 6.6	159.1 ± 7.9	0.19 ± 7.6	0.03	0.93	
Best Driver Distance (yards)						
PLA	260.0 ± 30.4	258.8 ± 29.6	−1.2 ± 18.6	0.04	0.82	0.04^§^
SD	269.9 ± 18.5	283.5 ± 23.1	13.6 ± 29.0	0.75	0.10	

Values are mean ± SD. ^§^= Group differences assessed using ANCOVA with respective baseline scores as the covariate due to significant difference between groups at baseline.

No significant group × time interaction effects were revealed for the following blood variables (Table 4): red blood cell count, hemoglobin, hematocrit, glucose, blood urea nitrogen (BUN), creatinine, BUN: creatinine, sodium, potassium, chloride, carbon dioxide, calcium, total protein, albumin, globulin, albumin: globulin, bilirubin, alkaline phosphatase, alanine aminotransferase, aspartate aminotransferase, total cholesterol, triglycerides, HDL cholesterol, VLDL cholesterol, LDL cholesterol, vitamin D (25-hydroxy D) and C-reactive protein. Only a small number of blood variables experienced changes or seemed to exhibit within-group changes over time. In all situations, the magnitude of change was within clinically accepted normative ranges for these variables. Specifically, in the SD group significant changes (p < 0.05) were noted in diastolic blood pressure, carbon dioxide, and total cholesterol while in the PLA group significant changes were noted in sodium, potassium, and glucose. Both groups experienced significant changes in total protein, globulin, and albumin: globulin ratio. No significant group × time interaction effects were reported for any of the pain indices, physical activity or enjoyment scales (Table 5, p > 0.05).

**Table 4:** **Hemodynamic, whole blood and clinical chemistry markers of health and safety**

dummyonly	**Intervention**			**Comparison of change (p)**		
	**Pre**	**Post**	**Change**	**Effect Size (** ***d*** **)**	**Within Groups**	**Group x Time**
Resting Heart Rate (beats/min)						
PLA	67.5 ± 8.4	66.8 ± 7.2	−0.7 ± 6.3	0.09	0.70	0.81
SD	61.4 ± 7.4	60.1 ± 6.2	−1.3 ± 6.2	0.19	0.45	
Systolic Blood Pressure (mm Hg)						
PLA	133.3 ± 15.0	125.9 ± 12.3	−7.4 ± 13.2	0.54	0.07	0.76
SD	129.6 ± 11.0	123.7 ± 10.2	−5.9 ± 12.2	0.56	0.10	
Diastolic Blood Pressure (mm Hg)						
PLA	81.5 ± 6.5	79.5 ± 6.6	−1.9 ± 5.6	0.16	0.22	0.10
SD	80.0 ± 6.9	73.0 ± 9.6	−7.1 ± 9.1	0.85	0.01*	
White Blood Cell Count (x10^6^/UL)						
PLA	5.6 ± 0.9	5.4 ± 1.4	−0.19 ± 1.4	0.13	0.62	0.55
SD	4.6 ± 1.4	4.7 ± 1.1	−0.14 ± 1.4	0.08	0.73	
Red Blood Cell Count (x10^6^/UL)						
PLA	5.3 ± 0.4	5.2 ± 0.4	−0.06 ± 0.21	0.40	0.30	0.83
SD	5.0 ± 0.3	5.0 ± 0.3	−0.05 ± 0.09	0.00	0.07	
Hemoglobin (grams/dL)						
PLA	15.7 ± 1.0	15.5 ± 1.0	−0.24 ± 0.6	0.20	0.16	0.20
SD	14.6 ± 0.9	14.7 ± 0.7	−0.57 ± 0.6	0.12	0.72	
Hematocrit (%)						0.96
PLA	46.0 ± 2.9	45.6 ± 2.9	−0.45 ± 1.9	0.14	0.42	
SD	43.3 ± 1.7	42.8 ± 1.9	−0.42 ± 0.9	0.28	0.10	
Glucose (mg/dL)						0.10
PLA	93.0 ± 5.7	87.1 ± 7.0	−5.8 ± 7.1	0.90	0.01*	
SD	88.9 ± 8.2	90.6 ± 11.4	−1.9 ± 15.0	0.20	0.64	
Blood Urea Nitrogen (mg/dL)						0.35
PLA	15.4 ± 3.7	15.1 ± 4.3	−0.31 ± 2.4	0.07	0.65	
SD	18.7 ± 6.3	19.2 ± 6.4	- 0.5 ± 2.0	0.08	0.37	
Creatinine (mg/dL)						0.28
PLA	1.01 ± 0.17	1.02 ± 0.16	0.00 ± 0.09	0.00	0.76	
SD	1.08 ± 0.11	1.13 ± 0.12	0.05 ± 0.11	0.00	0.11	
BUN:Creatinine						0.57
PLA	15.1 ± 2.6	14.7 ± 3.9	−0.38 ± 2.1	0.12	0.52	
SD	17.2 ± 5.0	17.3 ± 6.1	−0.07 ± 2.1	0.02	0.90	
Sodium (mEq/L)						0.47
PLA	139.5 ± 1.6	140.4 ± 1.2	0.92 ± 1.4	0.64	0.04*	
SD	139.7 ± 1.5	140.1 ± 1.1	0.43 ± 2.0	0.30	0.44	
Potassium (mEq/L)						0.14
PLA	4.2 ± 0.26	4.5 ± 0.19	0.31 ± 0.36	1.18	0.01*	
SD	4.3 ± 0.32	4.4 ± 0.34	0.11 ± 0.33	0.39	0.25	
Chloride (mEq/L)						0.32
PLA	102.3 ± 2.1	102.0 ± 1.1	−0.31 ± 1.7	0.18	0.52	
SD	102.3 ± 1.9	101.4 ± 1.7	−0.93 ± 1.5	0.50	0.04*	
Carbon Dioxide (mmol/L)						0.11
PLA	24.1 ± 1.4	24.5 ± 1.8	0.46 ± 1.8	0.25	0.36	
SD	24.8 ± 2.2	26.4 ± 2.0	1.57 ± 1.7	0.76	0.005*	
Calcium (mg/dL)						0.31
PLA	9.63 ± 0.34	9.68 ± 0.39	−0.05 ± 0.33	0.28	0.62	
SD	9.49 ± 0.31	9.39 ± 0.22	−0.1 ± 0.40	0.39	0.37	
Total Protein (g/L)						0.69
PLA	7.39 ± 0.34	7.08 ± 0.39	−0.31 ± 0.36	0.85	0.01*	
SD	6.96 ± 0.28	6.70 ± 0.28	−0.26 ± 0.30	1.00	0.007*	
Albumin (g/dL)						0.72
PLA	4.64 ± 0.30	4.65 ± 0.27	−0.01 ± 0.25	0.33	0.91	
SD	4.60 ± 0.16	4.58 ± 0.14	−0.02 ± 0.15	0.00	0.61	
Globulin (g/dL)						0.27
PLA	2.75 ± 0.23	2.43 ± 0.26	−0.32 ± 1.7	1.18	<0.001*	
SD	2.36 ± 0.26	2.12 ± 0.23	−0.24 ± 0.19	1.18	<0.001*	
Albumin: Globulin						0.62
PLA	1.70 ± 0.20	1.93 ± 0.24	0.23 ± 0.15	1.00	<0.001*	
SD	1.99 ± 0.25	2.19 ± 0.26	0.20 ± 0.17	0.67	<0.001*	
Bilirubin (mg/dL)						0.94
PLA	0.62 ± 0.25	0.60 ± 0.27	−0.02 ± 0.13	0.00	0.67	
SD	0.65 ± 0.21	0.63 ± 0.31	−0.02 ± 0.25	0.39	0.75	
Alkaline Phosphatase (U/L)						0.89
PLA	74.0 ± 22.6	74.2 ± 24.4	0.15 ± 12.2	0.01	0.96	
SD	67.1 ± 13.8	66.8 ± 14.8	--0.4 ± 5.3	0.02	0.81	
Aspartate Aminotransferase, AST (U/L)						0.59
PLA	25.9 ± 15.3	24.7 ± 13.6	−1.23 ± 6.23	0.08	0.49	
SD	28.0 ± 10.2	27.8 ± 9.5	−0.21 ± 2.83	0.02	0.78	
Alanine Aminotransferase, ALT (U/L)						0.66
PLA	31.2 ± 16.9	29.0 ± 16.3	−2.15 ± 7.6	0.13	0.33	
SD	27.3 ± 11.7	23.9 ± 8.8	−3.4 ± 6.5	0.33	0.07	
Total Cholesterol (mg/dL)						0.55
PLA	186.2 ± 48.0	182.2 ± 46.2	−3.9 ± 26.8	0.08	0.61	
SD	166.3 ± 40.6	157.3 ± 35.9	−9.0 ± 15.9	0.23	0.053*	
Triglycerides (mg/dL)						0.34
PLA	116.0 ± 51.7	115.8 ± 78.7	−0.23 ± 55.3	0.00	0.99	
SD	92.0 ± 84.3	71.8 ± 36.6	−20.2 ± 50.6	0.31	0.16	
HDL Cholesterol (mg/dL)						0.36
PLA	47.5 ± 9.4	47.2 ± 11.6	−0.31 ± 6.0	0.03	0.86	
SD	53.1 ± 12.9	50.9 ± 10.5	−2.21 ± 4.6	0.19	0.10	
VLDL Cholesterol (mg/dL)						0.33
PLA	23.2 ± 10.3	23.2 ± 15.7	0.00 ± 11.2	0.00	1.00	
SD	18.4 ± 17.0	14.3 ± 7.3	−4.14 ± 10.2	0.31	0.15	
LDL Cholesterol (mg/dL)						0.90
PLA	115.5 ± 41.6	111.9 ± 40.5	−3.62 ± 23.6	0.09	0.59	
SD	94.7 ± 31.4	92.1 ± 32.7	−2.64 ± 14.4	0.08	0.51	
Vitamin D (as 25(OH)D, ng/mL)						0.46
PLA	21.5 ± 5.2	23.3 ± 5.0	1.78 ± 3.94	0.35	0.13	
SD	35.1 ± 29.0	40.9 ± 45.4	3.95 ± 19.0	0.15	0.27	
C-Reactive Protein (mg/L)						0.64
PLA	1.41 ± 0.90	1.19 ± 0.65	−0.22 ± 0.6	0.25	0.19	
SD	1.46 ± 1.57	1.03 ± 1.11	−0.43 ± 1.5	0.36	0.29	

Values are mean ± SD. *= Significant within group change via t-test.

**Table 5:** **Pain and quality of life data**

dummyonly	**Intervention**			**Comparison of change (p)**		
	**Pre**	**Post**	**Change**	**Effect size (** ***d*** **)**	**Within groups**	**Group × Time**
Worst Pain						
PLA	1.62 ± 2.60	1.38 ± 2.14	−0.23 ± 1.09	0.08	0.46	0.52
SD	0.79 ± 1.31	0.93 ± 1.33	0.14 ± 1.79	0.08	0.77	
Least Pain						
PLA	0.31 ± 0.63	0.31 ± 0.85	0.00 ± 0.58	0.00	1.00	0.50
SD	0.36 ± 0.74	0.14 ± 0.53	−0.21 ± 0.97	0.49	0.43	
Average Pain						
PLA	1.38 ± 1,89	1.15 ± 1.52	−0.23 ± 0.83	0.12	0.34	0.97
SD	0.79 ± 1.12	0.57 ± 1.09	−0.21 ± 1.53	0.18	0.61	
Pain Level Now						
PLA	0.69 ± 1.44	0.69 ± 1.32	0.00 ± 1.68	0.00	1.00	0.48
SD	0.43 ± 0.94	0.07 ± 0.27	−0.36 ± 0.74	0.45	0.10	
Relief						
PLA	13.8 ± 34.0	6.2 ± 22.2	−7.7 ± 27.7	0.26	0.34	0.53
SD	16.4 ± 33.9	18.6 ± 37.0	−2.1 ± 49.0	0.06	0.87	
Current Activity						0.88
PLA	0.54 ± 1.13	0.31 ± 0.85	−0.23 ± 1.4	0.20	0.55	
SD	0.50 ± 1.34	0.36 ± 0.74	−0.14 ± 1.7	0.10	0.75	
Current Mood						0.45
PLA	0.31 ± 0.75	0.15 ± 0.38	−0.15 ± 0.69	0.16	0.44	
SD	0.14 ± 0.53	0.21 ± 0.60	−0.07 ± 0.83	0.18	0.75	
Walking						0.08
PLA	0.38 ± 1.12	0.85 ± 1.81	−0.46 ± 0.97	0.27	0.11	
SD	0.21 ± 0.43	0.14 ± 0.53	−0.07 ± 0.47	0.22	0.58	
Normal Work						0.67
PLA	0.31 ± 0.63	0.23 ± 0.60	−0.07 ± 0.76	0.17	0.72	
SD	0.43 ± 0.94	0.21 ± 0.58	−0.21 ± 0.89	0.26	0.39	
Sleep Quality						0.48
PLA	0.46 ± 1.1	0.45 ± 1.1	0.00 ± 1.0	0.00	1.00	
SD	0.43 ± 1.1	0.14 ± 0.4	−0.29 ± 1.1	0.36	0.34	
Yale P1						0.26
PLA	5471 ± 4118	5954 ± 4705	- 483 ± 2436	0.11	0.49	
SD	6376 ± 6967	5175 ± 2238	−1200 ± 4755	0.22	0.36	
Yale P2						0.77
PLA	85.5 ± 34.3	64.3 ± 26.1	−21.2 ± 45.6	0.70	0.12	
SD	114.9 ± 57.6	86.5 ± 52.5	−28.4 ± 76.8	0.52	0.19	
PCS						0.35
PLA	52.6 ± 7.1	56.0 ± 2.8	3.4 ± 5.7	0.63	0.05	
SD	53.9 ± 4.3	55.5 ± 2.7	1.6 ± 4.0	0.45	0.17	
MCS						0.59
PLA	55.5 ± 4.2	53.6 ± 6.5	−1.87 ± 6.58	0.35	0.33	
SD	56.1 ± 5.1	55.3 ± 3.5	−0.82 ± 2.85	0.18	0.30	

Values are mean ± SD. No significant changes between or within groups were noted.

## Discussion

Golf is a unique sport that requires a challenging combination of physical, mental and emotional attributes. Nutritional formulations developed to enhance these attributes are lacking, but multiple ingredients are available that have been examined scientifically which may improve golfing performance. The primary findings from the present study were that, over a 4-week period of supplementation, a blend of ingredients found in SD (e.g. creatine monohydrate, coffea arabica fruit extract [CoffeeBerry®], calcium fructoborate [FruiteX-B®], and vitamin D) significantly increased best drive distance and tended to improve average driver distance (p = 0.07) more so than the placebo group. In addition, both groups experienced significant improvements in bench press strength while the SD also experienced significant improvements in peak power and peak velocity production after the first set of bench press throws while no such changes were observed in the placebo group. Supplementation was well tolerated and no safety concerns/side effects were noted.

Although this study was not designed to determine the mechanisms underpinning any observed changes with SD supplementation, one or more of the ingredients in the formula were likely responsible for the observed changes. For example, a number of previous investigations using creatine monohydrate at the dosage provided in this study (10 grams/day × first two weeks, 5 grams/day × last two weeks) have routinely reported improvements in strength, upper-body performance, power and overall exercise capacity [3,7]. Thus, even though we did not collect muscle phosphagen data it is likely that the observed non-significant improvements in upper-body power and velocity primarily stemmed from the creatine monohydrate contained in SD. Future studies should utilize three groups to confirm this hypothesis (i.e. placebo vs. SD formula vs. creatine control).

Another potential candidate for performance enhancement in the SD formula is caffeine. As a dietary supplement, caffeine has successfully been used for years at doses of 3–6 mg/kg of body mass to improve both cognitive and physical aspects of exercise performance [10,11]. In the present study, no assessments of focus or concentration were made to evaluate changes as a result of supplementation because of the relatively low dose of caffeine in the test product (i.e. 50 mg or ~ 0.6 mg/kg body mass for these subjects). In addition, the last dose of SD (or placebo) was taken 24-hours prior to day 30 testing. Thus, we consider it unlikely that the caffeine contained in the *coffea arabica* fruit extract (CoffeeBerry®) of SD played a major role in the results. It is worth mentioning we cannot completely discount the potential effects that other unique ingredients in CoffeeBerry® (e.g., various chlorogenic acids and plant phenolics), may have had on these results.

Finally, data surrounding boron and vitamin D continue to lend support towards these nutrients and their ability to mitigate pain [20] as well as improve bone [21] and muscle health [23]. Findings from the present study did not reveal improved outcomes associated with self-reported pain, fatigue or weakness levels as assessed before and after the four week supplementation period. We speculate that the primary reason for these findings was associated with the existing joint and bone health of our study participants. For example, our study participants were otherwise healthy, middle-aged men who golfed on a regular basis and as a result were maintaining a basic level of physical activity. When compared to the study participants in other published trials showing improvements in self-reported pain [19–21,24], participants from the present study were younger, healthier and had minimal baseline levels of pain. The lack of change found in serum levels of 25-hydroxy vitamin D as a result of supplementation was not entirely unexpected. Given the moderate dose (1,000 IU/day), the time of year the study was conducted (late Fall), and general latitude of the study location (Kent, OH = 41.15^°^ N), it is our contention that the majority of supplemented vitamin D was quickly absorbed by peripheral tissues.

From an adverse event and/or clinical safety perspective, the SD supplement was well tolerated with no significant group × time interactive changes in any of the measured clinical markers (Tables 4 and 5). A few variables did experience significant within-group changes (e.g., diastolic blood pressure, glucose, sodium, potassium, carbon dioxide, albumin: globulin ratio, total protein and globulin, etc.), but in all such circumstances, both groups changes remained within clinical accepted normative values.

The strengths of the present study are that it was a double-blind, placebo-controlled investigation using a “free-living” approach. Thus, the findings are applicable to many consumers who golf but do not follow a rigid diet or exercise program. To date, extremely limited research is available exploring the potential impact of various nutritional agents on golf performance, with one of the only published accounts reporting an improvement in stress hormone levels and shot-making after phosphatidylserine supplementation [2]. The disadvantages of the present study primarily revolve around the pilot nature of the investigation and the lack of female subjects. The authors recognize that the relatively small number of subjects and short supplementation period (4 weeks) likely hindered the ability to uncover significant effects in some outcome measures, particularly since the participants were relatively young and healthy and were not required to follow a particular exercise training program or dietary regimen. It is also important to discuss other considerations related to our data and findings. For example, the positive outcome surrounding best drive distance is likely impacted by a number of other factors that we weren’t able to be measured in our study design including, but not limited to: club head acceleration at the point of impact with the ball, club head deceleration through the zone of impact, rotational power, improved accuracy of club head placement relative the ball, club face angle at ball strike, and musculoskeletal range of motion. Two additional factors are upper-body strength and power that were measured in the present study by determining bench press 1RM and sagittal plane power development during bench press throws. The authors recognize that performance of the bench press exercise may be viewed by some as having very little carryover to golf swing performance, but these parameters were chosen due to their high reliability of measurement and as being valid and commonly used measures of maximal strength and power in the sports science literature. Additionally, it is important to highlight that Marta and colleagues indicate that the pectoralis major, deltoid, and latissimus dosi are the most active muscle groups during the golf swing movement, particularly during the acceleration phase [29]. A three-dimensional analysis of golf swing performance using two common clubs was employed as a measurement method of golfing performance. While much more practical, this measurement method only reports on final performance and thus does not allow for a clearer understanding of what aspects of performance SD may have improved according to our findings, particularly as they relate to best drive distance. In this respect, it is important for the reader to understand other factors may have impacted our observed outcomes related to best driver distance.

## Conclusions

This preliminary investigation yielded significantly greater improvements in best drive distance (~13.6 yards) and a tendency for average driver distance to improve in healthy male golfers consuming SD for four weeks. Careful interpretation of these data is encouraged due to their preliminary nature and need to be followed-up with a larger and longer investigation.
